# State-specific cigarette use rates among service members and veterans, United States, 2017

**DOI:** 10.18332/tpc/111536

**Published:** 2019-09-03

**Authors:** Justin T. McDaniel, Robert Klesges

**Affiliations:** 1 Department of Public Health and Recreation Professions, Southern Illinois University, Carbondale, United States; 2 Center for Addiction and Prevention Research, Department of Public Health Sciences, University of Virginia, Charlottesville, United States

**Keywords:** military, veterans, cigarettes, tobacco, civilians, geography

## Abstract

**INTRODUCTION:**

Little is known about the geographical distribution of cigarette use among military service members and veterans. In this study, we estimated state-specific rates of current cigarette use for service members and veterans and compared these to the current cigarette use rates of civilians.

**METHODS:**

We used data from the 2017 Behavioral Risk Factor Surveillance System to generate survey-weighted percentages with 95% confidence intervals of current cigarette use among service members and veterans (SMVs) and civilians. Respondents (n=450016) were classified as an SMV if they answered in the affirmative to the following question: ‘Have you ever served on active duty in the United States Armed Forces, either in the regular military or in a National Guard or military reserve unit?’. Current cigarette users were persons who reported having smoked 100 cigarettes in their lifetime and smoked ‘some days’ or ‘every day’ at the administration of the survey.

**RESULTS:**

Nationally, 17.3% (95% CI: 16.6–18.0) of SMVs reported current cigarette use, while 16.2% (95% CI: 16.0–16.5) of civilians reported current cigarette use. By state, current cigarette use rates ranged from 10.0% in Utah (95% CI: 7.5–12.5) to 23.7% in Indiana (95% CI: 20.9–26.5) among service members and veterans, and from 8.8% in Utah (95% CI: 8.0–9.6) to 27.0% in West Virginia (95% CI: 25.3–28.6) among civilians.

**CONCLUSIONS:**

Resources and interventions directed at cigarette smoking cessation should consider military status and geography when recruiting participants.

## INTRODUCTION

Nationally representative studies in the United States have shown that cigarette use rates are higher among service members and veterans (SMVs) than for civilians^1^. However, no study has investigated the state-level distribution of cigarette use among SMVs and civilians. Because the United States Veterans Health Administration spends approximately $2 billion per year in healthcare directly attributable to smoking^2^, there is a need for the identification of high cigarette use regions and subsequent development of geographically targeted programming. As such, the aim of the present study was to estimate the rate of current cigarette use among SMVs and civilians, separately, for each state in the United States.

## METHODS

State-specific estimates of self-reported current cigarette use among SMVs and civilians were calculated using data from the 2017 Behavioral Risk Factor Surveillance System (BRFSS)^3^. The BRFSS is a nationally representative telephone-based survey conducted annually in the United States by the Centers for Disease Control and Prevention. Information is collected from residents in all 50 states regarding their health behaviors, use of preventive health services, and chronic health conditions.

Respondents (n=450016) in this study were classified as an SMV if they answered in the affirmative to the following question: ‘Have you ever served on active duty in the United States Armed Forces, either in the regular military or in a National Guard or military reserve unit?’. Responses were: yes=57868, no=391408, don’t know=120, refused=609, and missing=11. Respondents who answered ‘don’t know’ or ‘refused’ were excluded from the analysis.

Two questions were used to determine current cigarette use: a) have you smoked at least 100 cigarettes in your entire life; and b) do you now smoke cigarettes every day, some days, or not at all?. Current cigarette users were persons who reported having smoked 100 cigarettes and smoked ‘some days’ or ‘every day’ at the administration of the survey. Respondents who answered ‘don’t know/ refused’ to the first smoking question were excluded from the analysis. In total, 15934 respondents were excluded because of missing data on the ‘current cigarette user’ variable, thus the final sample size was 433342.

National and state-specific estimates of and 95% confidence intervals for current cigarette use were adjusted for the BRFSS complex survey design. Sampling weights accounted for survey non-coverage, non-response, and telephone-only households^4^. Estimates for states with unweighted sample sizes of SMVs or civilians <50 or relative standard errors (RSE) >30% were suppressed^5^. For each state, we also regressed - in logistic regression models - current cigarette use (0=no, 1=yes) on SMV status (0=no, 1=yes) in order to obtain a p-value for differences in smoking prevalence between civilians and SMVs.

## RESULTS

Among 55868 (weighted n=25285001) SMVs in the United States, 8313 (weighted n=4362402) reported current use of cigarettes (17.3%, 95% CI: 16.6–18.0). Among 375080 (weighted n=217077890) civilians in the United States, 55071 (weighted n=35211188) reported current use of cigarettes (16.2%, 95% CI: 16.0–16.5). The difference in current cigarette smoking rates at the national level between SMVs and civilians was statistically significant at an alpha level of 0.05.

By state (Table 1), current cigarette use rates ranged from 10.0% (Utah) to 23.7% (Indiana) among SMVs, and from 8.8% (Utah) to 27.0% (West Virginia) among civilians. Based on an alpha level of 0.05, results showed that 4 states/districts (i.e. Arizona, California, District of Columbia, and Idaho) exhibited higher current cigarette use rates among SMVs than among civilians, while 3 states (i.e. Delaware, Florida, and West Virginia) exhibited higher current cigarette use rates among civilians than among SMVs. Differences in current cigarette smoking rates between civilians and SMVs were not significant in all other states.

**Table 1:** State-specific rates of current cigarette use among adults with no military experience and adult service members and veterans, based on data from the Behavioral Risk Factor Surveillance System (BRFSS), United States, 2017

	*No Military Experience*				*Service Member or Veteran*				
				*95% CI*				*95% CI*		
	n*	*%^†^*	*Low*	*High*	n*	*%^†^*	*Low*	*High*	*p^§^*
**United States**	55071	16.20	16.00	16.50	8313	17.30	16.60	18.00	0.01
**State**									
Alabama	5569	21.11	19.57	22.65	898	19.96	16.27	23.64	0.58
Alaska	2534	20.89	17.97	23.82	547	21.66	15.22	28.10	0.83
Arizona	12404	15.23	14.36	16.10	2340	17.98	15.90	20.05	0.01
Arkansas	4362	22.71	20.24	25.18	730	18.48	13.33	23.63	0.17
California	7844	11.02	10.02	12.02	859	14.36	11.03	17.68	0.04
Colorado	7775	14.65	13.63	15.68	1141	14.64	12.07	17.20	0.99
Connecticut	9019	12.84	11.79	13.90	1040	11.28	8.33	14.24	0.35
Delaware	3360	17.67	15.81	19.54	594	12.66	8.84	16.48	0.04
DC	3410	13.95	12.40	15.50	335	19.67	14.46	24.89	0.02
Florida	17838	16.54	15.26	17.83	3170	13.11	10.58	15.64	0.03
Georgia	4887	17.14	15.70	18.58	830	19.41	15.83	22.99	0.23
Hawaii	6352	12.69	11.53	13.84	1067	13.41	10.34	16.48	0.66
Idaho	4125	13.78	12.25	15.30	618	18.50	13.93	23.07	0.04
Illinois	4794	15.49	14.09	16.89	551	15.11	11.37	18.84	0.85
Indiana	11624	21.57	20.50	22.64	1626	23.70	20.90	26.50	0.15
Iowa	6543	16.84	15.72	17.96	888	18.93	15.75	22.11	0.21
Kansas	18018	17.30	16.56	18.04	2654	17.87	15.99	19.74	0.58
Kentucky	7369	24.96	23.27	26.65	970	22.07	18.07	26.08	0.21
Louisiana	3983	23.41	21.62	25.20	547	19.73	15.28	24.17	0.15
Maine	8020	17.13	15.75	18.51	1297	18.11	14.61	21.62	0.60
Maryland	11093	13.66	12.59	14.73	1832	15.38	12.34	18.42	0.28
Massachusetts	5962	13.40	11.98	14.81	696	17.27	11.96	22.58	0.13
Michigan	9347	19.06	17.98	20.14	1139	21.17	18.06	24.27	0.20
Minnesota	14451	14.42	13.63	15.20	1951	15.31	13.18	17.44	0.43
Mississippi	4159	22.70	20.71	24.68	648	18.42	14.22	22.62	0.09
Missouri	6380	20.89	19.42	22.36	987	19.74	15.87	23.61	0.59
Montana	4915	17.37	15.84	18.90	865	15.75	12.24	19.27	0.42
Nebraska	13013	15.67	14.64	16.69	1828	13.06	10.76	15.37	0.06
Nevada	3077	16.76	14.74	18.79	563	22.77	16.18	29.36	0.06
New Hampshire	4706	16.03	14.31	17.76	787	12.63	8.60	16.65	0.16
New Jersey	10110	13.56	12.39	14.72	1092	16.46	12.23	20.68	0.17
New Mexico	5398	17.84	16.30	19.37	850	14.85	11.60	18.10	0.12
New York	10524	13.89	12.95	14.83	1016	16.63	12.95	20.31	0.13
North Carolina	4044	17.18	15.58	18.78	693	17.04	13.50	20.57	0.94
North Dakota	5838	17.86	16.46	19.26	901	21.37	17.64	25.11	0.07
Ohio	10401	21.25	19.99	22.52	1465	20.36	17.29	23.42	0.60
Oklahoma	5393	20.23	18.70	21.75	972	19.74	16.43	23.05	0.79
Oregon	4453	16.28	14.93	17.63	663	14.61	11.34	17.89	0.37
Pennsylvania	5626	18.67	17.29	20.04	721	19.66	15.86	23.45	0.63
Rhode Island	4664	15.21	13.55	16.86	715	12.64	9.27	16.01	0.20
South Carolina	9225	18.93	17.73	20.13	1654	17.60	15.04	20.16	0.36
South Dakota	5869	19.08	16.98	21.17	964	20.69	15.64	25.73	0.56
Tennessee	4826	22.68	20.92	24.45	735	22.04	17.80	26.28	0.79
Texas	10120	15.42	13.89	16.96	1556	18.27	13.77	22.76	0.22
Utah	8961	8.79	8.01	9.56	996	10.00	7.48	12.51	0.35
Vermont	5484	15.68	14.28	17.08	722	17.10	12.56	21.64	0.55
Virginia	7727	16.15	14.95	17.34	1546	17.88	15.10	20.66	0.25
Washington	10873	13.52	12.63	14.42	1888	13.54	11.47	15.61	0.99 <
West Virginia	4650	26.95	25.27	28.63	717	19.07	15.60	22.54	0.001
Wisconsin	4883	16.03	14.58	17.47	651	16.23	12.21	20.25	0.93
Wyoming	3707	18.75	17.05	20.45	654	18.63	14.78	22.48	0.96

* Unweighted sample size. CI: confidence interval.

† Weighted %; all relative standard errors (RSE) for percentages <30%.

§ p-value from z-statistic for SMV coefficient in logistic regression model.

## DISCUSSION

Nationally aggregated data from the 2017 BRFSS indicated that current cigarette smoking rates were 6.4% higher among SMVs than civilians. However, significant state-level variation was observed in cigarette use rates in these two populations, with rates as high as 23.7% among SMVs (Indiana) and 27.% among civilians (West Virginia).

While previous studies have concluded that SMVs have higher cigarette use rates at the national level, these studies did not examine cigarette use rates at finer geographical levels (i.e. the state level). The results of this study could inform state-based efforts to prevent and end cigarette use, especially among SMVs. Specifically, one possible policy-related intervention to eliminate cigarette smoking among active duty military service members would include a comprehensive ban on tobacco use at all stages of military service. A current military policy prohibits tobacco use during basic training; however, a ban on tobacco use throughout the entire length of military service would be more effective^6^.

Other state-level policy interventions to curb cigarette use among SMVs — veterans in particular — could include higher excise taxes on cigarettes. Yet another state-level policy intervention in high cigarette use states could center on the development of tailored messaging strategies for SMVs via state quitlines. Although the United States Department of Veterans Affairs (VA) has a quitline for veterans specifically, non-VA quitlines could incorporate veteran specific response strategies, as not all veterans use VA-related resources. Given that many state quitlines maintain specialized materials for certain populations, such as sexual minorities, individuals with low literacy levels, pregnant women, senior citizens, and certain racial/ethnic minorities^7^, incorporation of SMV specific materials could result in more effective cessation efforts for SMVs who use non-VA state quitlines.

Finally, cigarette smoking cessation among military veterans may be aided by veteran service organizations, such as The Mission Continues and Team Red, White, and Blue. These veteran service organizations exist to help veterans reintegrate into civilian society by providing social connection opportunities and encouraging a healthy lifestyle, among other things, following military service^8,9^. These veteran service organizations maintain chapters in many states in the United States. Chapters operating in states with high SMV cigarette use rates may consider implementing cigarette smoking cessation campaigns to curb the excessive smoking rate.

### Strengths and limitations

Strengths of this study include the large, nationally representative, geo-coded sample as well as the provision of a dataset (Table 1) that could be used by healthcare providers and legislators responsive to the needs of SMVs. Limitations of this study include the lack of temporal analysis and the use of self-reported data.

## CONCLUSIONS

This is the first study to estimate the distribution of cigarette use rates among SMVs in all 50 states in the United States. Significant state-level variation was observed in cigarette use rates between SMV and civilian populations. Resources and interventions directed at cigarette smoking cessation should, therefore, consider military status and geography when recruiting participants. Future studies should consider estimating cigarette use rates among SMVs at finer geographical resolutions, similar to the way in which mental distress rates were estimated at the county level in the McDaniel et al.^10^ study of Kentucky SMVs. Local-level estimates could be used to develop unique interventions tailored specifically for a small geographical location.
